# Childhood Trauma and Adult Psychological Adjustment: Resilience in Older Adult Survivors of Institutional Abuse

**DOI:** 10.1093/geroni/igaa057.1508

**Published:** 2020-12-16

**Authors:** Shauna Mc Gee, Andreas Maercker, Alan Carr, Myriam Thoma

**Affiliations:** 1 University of Zürich, Zürich, Switzerland; 2 University College Dublin, Dublin, Ireland

## Abstract

Several international cohorts of older adults share past experiences of welfare-related adversity. In Ireland, reports of childhood maltreatment, neglect, and abuse within institutional welfare settings included a harsh regime, childhood labour, and physical and sexual assault. Preliminary research with these Irish survivors revealed a high prevalence of psychological disorders in adulthood. A pathological perspective of aging is often applied to such older adults, due to the long-term health consequences associated with childhood trauma. However, little is known about later life resilience or resilience mechanisms in this population. Therefore, using conceptual models of resilience, this qualitative study aimed to investigate factors associated with resilience in older adult survivors of childhood institutional abuse. Participants were 17 Irish older adults, 50-77 years of age, with experiences of childhood abuse in institutional care settings. Semi-structured interviews were conducted, lasting 60-120 minutes. Transcribed interviews were analysed using the Framework Analysis method. Nine themes were derived from the data, including core, internal, and external resilience factors: Individual characteristics, personality characteristics, support systems, goal attainment, adaptive belief systems, processing, influential events and experiences, recognition and collective identity, and access to services. Results suggest that resilience can be understood not only as an inherent trait, but also as a learnable set of behaviours, thoughts, and attitudes, which can be supported by external resources in an older adults’ environment. The identification of personal and contextual factors underpinning resilience in older adults with trauma experiences may help foster a more positive, strengths-based approach to aging in psychological research and practice.

